# Macrophages: their role in immunity and their relationship with fatty acids in health and disease

**DOI:** 10.3389/fimmu.2025.1694892

**Published:** 2025-12-02

**Authors:** Mayte Rueda-Munguía, Luis Alberto Luévano-Martínez, Gerardo García-Rivas, Elena Cristina Castillo, Omar Lozano

**Affiliations:** 1 Escuela de Medicina y Ciencias de la Salud Tec Salud, Tecnologico de Monterrey, Monterrey, Mexico; 2 Institute for Obesity Research, Tecnologico de Monterrey, Monterrey, Mexico

**Keywords:** macrophages, fatty acids, inflammation, metabolic diseases, immunometabolism

## Abstract

The intricate interplay between macrophage biology and lipid metabolism has emerged as a critical determinant of metabolic homeostasis, disease progression and pathogenesis. This comprehensive review explores the molecular mechanisms through which fatty acids activate macrophage function, emphasizing their selective engagement of pattern recognition receptors such as Toll-like receptors (TLRs), CD36, and GPR120. Notably, saturated fatty acids (SFAs) like lauric acid (C12:0) and palmitic acid (C16:0) activate TLR2 and TLR4 signaling pathways. Palmitic acid triggers mitochondrial dysfunction and lysosomal destabilization, leading to NLRP3 inflammasome activation and chronic low-grade inflammation. In contrast, ω-3 polyunsaturated fatty acids (PUFAs), such as docosahexaenoic acid, help resolve inflammation through GPR120-mediated signaling and the production of specialized pro-resolving mediators (SPMs) like resolvins, protectins, and maresins. This review establishes a paradigm for understanding the complex relationship between dietary lipids, innate immunity, and metabolic health, with broad implications for immunometabolic interventions.

## Introduction

The immune system, an intricate network of cells including macrophages, neutrophils, dendritic cells, and natural killer (NK) cells (1, 2) relies significantly on the multifaceted functions of their cells to maintain systemic homeostasis (3). Of particular interest are macrophages, which constitute approximately 10% of immune cells (4), exhibit remarkable heterogeneity and plasticity (5), enabling them to play pivotal roles in both promoting and resolving inflammation.

Macrophages can be broadly classified into two categories: monocyte-derived macrophages, which originate from the bone marrow, circulate in the bloodstream, and subsequently extravasate into tissues, where they differentiate into macrophages; and tissue-resident macrophages, such as alveolar macrophages in the lungs and Kupffer cells in the liver, which emerge during fetal development from precursors in the yolk sac and fetal liver precursors (6). Unlike monocyte-derived macrophages, tissue-resident macrophages sustain their population through local proliferation, allowing them to adapt and respond specifically to their microenvironment (7).

Depending on external stimuli, macrophages polarize into distinct phenotypes, each performing specialized functions within tissues (8). The two primary phenotypes are pro-inflammatory macrophages (M1), which are activated in response to stimuli such as lipopolysaccharides (LPS) and cytokines like INF-γ (9), and the anti-inflammatory macrophages (M2), which are activated in the presence of interleukin (IL)-4 and IL-13, that play key roles in inflammation resolution, tissue repair, and homeostasis maintenance (10).

Beyond their traditional immune functions, macrophages are increasingly recognized as key regulators in metabolic diseases. In this context, fatty acids, long known for their roles as energy substrates and structural components, exert significant influence on macrophage polarization and function by activating or suppressing key signaling pathways (11). In metabolic disorders, macrophages often exhibit a mixed phenotype, distinct from classical M1 and M2 polarization, referred to as metabolically activated (MA) macrophages (12).

In addition to the classical M1 and M2 polarization spectrum, single-cell transcriptomic studies have identified a metabolically specialized subset of macrophages termed lipid-associated macrophages (LAMs). These cells mostly arise in adipose tissue during obesity and are characterized by the expression of genes associated with lipid metabolism, including TREM2, CD9 and lipoprotein lipase (LPL) (13). LAMs localize around hypertrophic or necrotic adipocytes, where they phagocytose lipids and cellular debris, hence fulfilling a protective and homeostatic function during initial metabolic stress. However, under conditions of chronic obesity, their sustained activation leads to the production of pro-inflammatory cytokines and maladaptive tissue remodeling, ultimately exacerbating metabolic dysfunction (14). Collectively, LAMs connect metabolism and immunology by merging lipid handling with inflammatory signaling, positioning them as a central component of the immunometabolic framework of adipose tissue.

**Figure 1:**
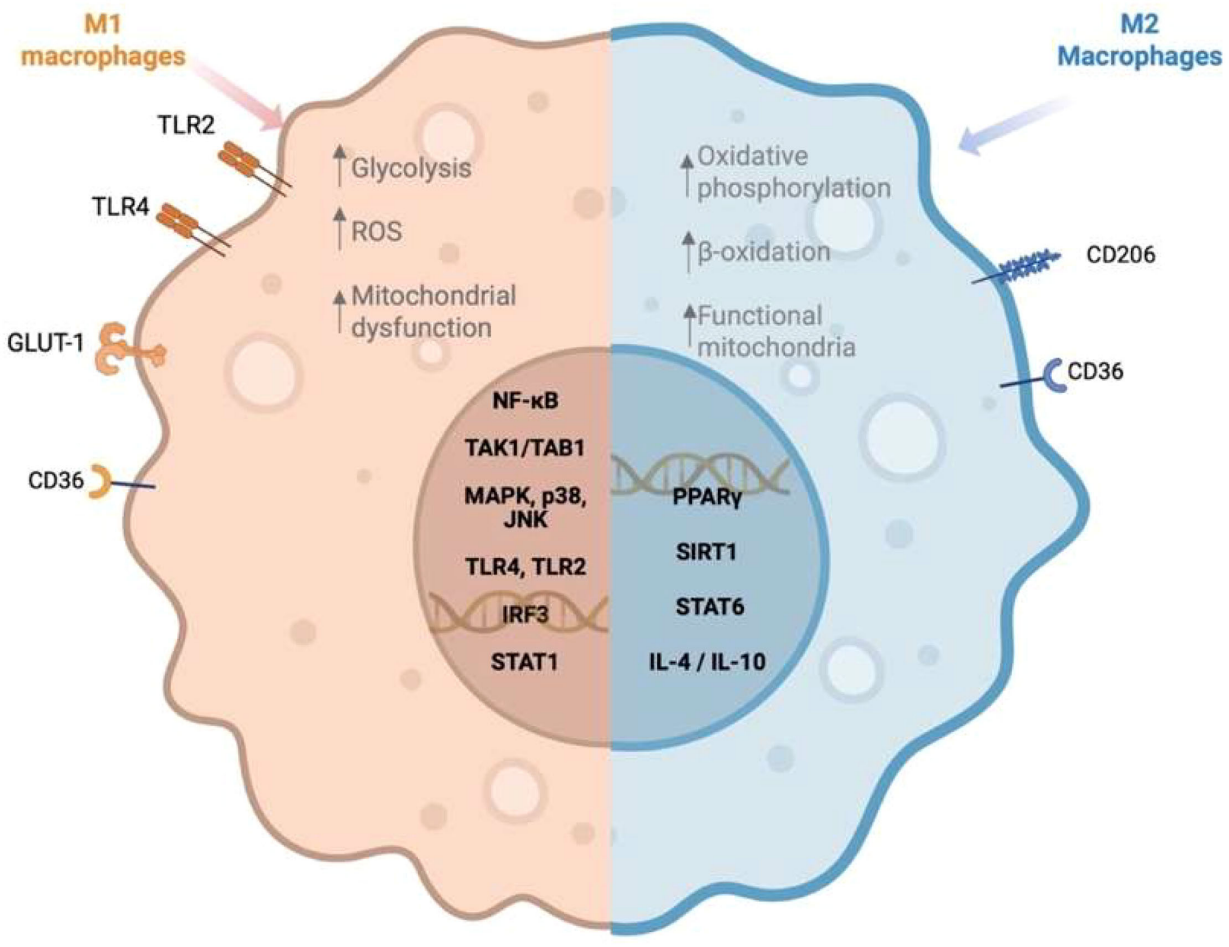
Macrophage polarization in response to saturated and unsaturated fatty acids. Created in https://BioRender.com.

In the context of a diet rich in animal fats and processed foods, oversupply of saturated fatty acids (SFAs) within tissues leads to their accumulation, particularly in adipose tissue, where macrophages accumulate excess fatty acids (15). This accumulation of SFAs influences macrophages’ function beyond their metabolic contributions as energy resources, leading to a MA phenotype (**Figure 1**). MA macrophages activate M1-like inflammatory pathways, promoting low-grade chronic inflammation and contributing to the pathogenesis of metabolic disorders (16, 17). The disruption in the function of macrophages occurs not only in the adipose tissue but also in tissue-resident macrophages, such as Kupffer cells in the liver and microglia in the brain (6). This systemic impact may drive chronic inflammation and increase susceptibility to a range of metabolic disorders, including obesity, diabetes type II, and cardiovascular diseases (18, 19).

Dietary SFAs, such as palmitic acid and lauric acid (C:12), activate Toll-like receptor 4 (TLR4) signaling pathways, leading to the activation of nuclear factor-kappa B (NF-κB) and the subsequent release of pro-inflammatory cytokines, including tumor necrosis factor-alpha (TNF-α), IL-1β, and IL-6, a pleiotropic cytokine associated with insulin resistance (20, 21). In addition, palmitic acid induces mitochondrial dysfunction and lysosomal destabilization, culminating in the activation of the NLRP3 inflammasome, a multiprotein complex strongly associated with obesity and type II diabetes (22). Caspase-1, a key component of the NLRP3 inflammasome, facilitates the maturation and activation of IL-1β, thereby amplifying the inflammasome response (23). However, evidence suggests that other inflammasomes, such as NLRC4, though less well characterized in the context of obesity, also contribute to IL-1β production and the exacerbation of inflammatory states (23).

Conversely, M2 macrophage polarization, driven by cytokines like IL-4, IL-13, IL-10, and transforming growth factor beta (TGF-β), serves a compensatory role in controlling inflammation, promoting tissue repair, and maintaining metabolic homeostasis (9). Certain unsaturated fatty acids (UFAs), such as ω-3 fatty acids like eicosapentaenoic acid and docosahexaenoic acid, have been shown to support M2 polarization and exert anti-inflammatory responses, counteracting the pro-inflammatory effects of SFAs (24). Notably, IL-10 released from M2 macrophages suppresses mechanistic target of rapamycin complex 1 (mTORC1) signaling, a pathway known to regulate glycolysis and mitochondrial respiration in immune cells, thereby reducing inflammation and oxidative stress (25). Additionally, TGF-β plays a crucial role in regulating adipogenesis, T cell differentiation, and mitochondrial metabolism in adipose tissue, further supporting metabolic homeostasis (26). These cytokines collectively counteract immune and metabolic dysfunction in obesity and insulin resistance, partly by mitigating the mitochondrial dysfunction and lysosomal destabilization induced by SFAs like palmitic acid. This shift toward M2 polarization and the anti-inflammatory phenotype supports metabolic health, particularly in counteracting chronic inflammation induced by diet-related factors.

In the context of metabolic disorders, the widespread dietary shift towards ultra-processed foods (UPFs), characterized by high SFA and deficiency in essential nutrients, exacerbates metabolic dysregulation (27). UPFs activate inflammatory pathways, including TLR4-NF-κB signaling, which directly impacts immune cell function, particularly macrophages (28). This signaling cascade skews macrophage polarization toward a MA phenotype, contributing to chronic low-grade inflammation and insulin resistance (29).

The excess SFAs present in UPFs also promote mitochondrial dysfunction and lysosomal destabilization in macrophages, further amplifying the inflammatory response by activating NLRP3 inflammasomes (30). These alterations in macrophage function, driven by the dietary intake of UPFs, are closely linked to the increased risk of obesity, type II diabetes, and cardiovascular diseases. Furthermore, epidemiological studies show that each 20-gram increase in UPF consumption is associated with a significant increase in serum cholesterol (TC) (β = 1.214; 95% CI:.159-2.269) and a reduction in high-density lipoprotein (HDL) (β = -0.371; 95% CI: -0.675 to -0.067), both of which contribute to dyslipidemia and promote a pro-inflammatory environment, exacerbating metabolic imbalance (27). These findings underscore the detrimental impact of UPFs on macrophage function and their role in driving immune dysfunction and metabolic disorders.

This review aims to elucidate and analyze the multifaceted interactions between macrophages and fatty acids and their contributions to the pathogenesis of metabolic and cardiovascular diseases. By bridging cellular mechanisms with dietary influences, we provide a comprehensive framework for understanding the immuno-metabolic dysregulation underpinning these conditions while identifying potential therapeutic targets.

For the compilation of the presented information in this review, keyword searches employing Boolean operators, such as “macrophages AND metabolic diseases,” “macrophages AND diabetes OR obesity,” “macrophages AND fatty acids,” and “fatty acids AND inflammation,” were used. A literature search was conducted across databases, including PubMed and ScienceDirect.

## Impact of SFA on the function of macrophages

A defining component of the modern Western diet is SFAs, which have been implicated in the rising global prevalence of metabolic diseases (31). SFAs are a class of lipids characterized by their fully saturated molecular structure, lacking double bonds between carbon atoms. These fatty acids are abundant in animal-derived products such as meat and dairy, as well as in certain tropical oils, including coconut and palm oil (32).

Fatty acids serve as crucial substrates for triglyceride (TG) storage and membrane formation, with their metabolism intricately linked to cellular energy balance. While TG serves as a physiological adaptation to excess energy intake, chronic overconsumption of SFAs disrupts lipid homeostasis, leading to ectopic TG accumulation in non-adipose tissues, a phenomenon known as lipotoxicity (30). Lipotoxic stress induces cellular dysfunction, particularly in hepatocytes, cardiomyocytes, and macrophages, where excessive SFAs are metabolized into bioactive lipids such as ceramides and diacylglycerols (DAGs) (33). These lipid intermediates disrupt insulin signaling and predispose macrophages to an inflammatory phenotype (11).

Exposure to SFAs drives macrophage polarization toward a pro-inflammatory M1-like phenotype. Specifically, palmitic acid (C16:0) triggers TLR signaling, activating the NF-κB and c-Jun N-terminal kinase (JNK) pathways (19). This signaling cascade primes macrophages to secrete cytokines such as TNF-α and IL-1β, contributing to the chronic low-grade inflammation characteristic of metabolic diseases (19). In addition to palmitic acid, other SFAs, including myristic acid (C14:0), lauric acid, and stearic acid (C18:0), have also been shown to induce mitochondrial dysfunction and impair oxidative phosphorylation, leading to a disruption of normal cellular energy metabolism (16). These alterations lead to increased production of reactive oxygen species (ROS), contributing to metabolic stress and promoting insulin resistance (34).

Additionally, both saturated and unsaturated fatty acids can be taken up through the scavenger receptor CD36, which also mediates the uptake of oxidized-low density lipoproteins (oxLDL) (35, 36). In the context of SFAs, CD36-mediated lipid accumulation impairs cholesterol efflux and promotes foam cell formation, thereby amplifying pro-inflammatory signaling (37, 38). Collectively, these SFA-induced changes in macrophage function accelerate metabolic dysfunction, reinforcing the link between dietary composition and the pathogenesis of metabolic disorders, including type II diabetes and cardiovascular diseases (34).

Findings summarized in **Table 1** highlight the interactions between various fatty acids and key metabolic pathways, demonstrating their impact on macrophage inflammation and metabolism. Palmitic acid activates mitogen-activated protein kinase (MAPK) and TLR4 pathways, leading to increased secretion of monocyte chemoattractant protein-1 (MCP-1) and promoting inflammatory responses via extracellular signal-regulated kinases 1/2 (ERK1/2) and TLR2/4 activation (19, 39). This pro-inflammatory effect is further amplified when palmitic acid is combined with palmitoleic acid (C16:1) (40). Notably, palmitoleic acid, frequently exerts opposing, anti-inflammatory actions. Experimental evidence shows that palmitoleic acid attenuates high-fat diet-induced proinflammatory macrophage polarization via AMPK activation (40) and inhibits palmitic acid-induced macrophage activation, thereby mitigating skeletal muscle insulin resistance in co-culture models of J774 macrophages and C2CL12 myotubes (41). Nonetheless, its protective effects are context-dependent: they are more pronounced at physiological concentrations (≤50 μM) and in macrophage or adipose tissue models, whereas in microglial cells, palmitoleic acid provides limited protection against palmitate-induced inflammation (42).

**Table 1:** Impact of SFAs on macrophages’ metabolic pathways and their effects.

Fatty acid	Metabolic pathways or associated receptors	Type of macrophages	Main results	Reference
Palmitic acid + LPS	TLR4, MAPK (JNK, ERK, p38)	RAW264.7	↑ MCP-1 transcription/secretion; ↑ TLR4 expression.	(19)
Palmitic acid + LPS	ERK1/2, ER stress	RAW264.7 and primary mouse hepatocytes	↑ mRNA levels TNF-α, IL-6, IL-1β, MCP-1	(21)
Palmitoleic (C16:1) acid vs. palmitic acid	TLR2, TLR4	J774A.1	↑ Proinflammatory state induction; TLR2 or TLR4 activation.	(40)
Palmitic acid	–	Lung macrophages from BALB/c mice	↑ Lung macrophages increase; ↑ Neutrophilic airway inflammation; MCP-1 induction; ↑ IL-1β, TNF-α production.	(52)
Palmitic acid vs. palmitoleic acid	P38, MAPK, JKN, TNF-α	J774, C2C12 myotubes	↑ Insulin resistance; ↑ p38 MAPK, JNK activation; ↑ Insulin sensitivity.	(41)
Stearic acid	Nuclear retinoid acid receptor (RAR), E-FABP	CD11c+ macrophages	↑ CD11c expression; ↑ obesity-associated inflammation.	(34)
Stearic acid, LPS, free fatty acids	AMPK, SIRT1, NF-κB	RAW264.7 and adipose tissue macrophages	↓ AMPK signaling by FFAs/LPS, phosphorylation of acetyl-CoA carboxylase	(43)
Stearic acid + TNF-α	TLR4, TBK1/IRF3	human monocytic cells	↑ MIP-1a, CCL3, and TLR4 blocking reduces the effect.	(37)
Lauric acid	GLUT-1, GLUT-3, mitochondrial biogenesis	Insulin-resistant THP-1 macrophages	↑ Glucose uptake improvement; ↑ Mitochondrial function; ↑ TFAM.	(45)
Lauric acid + INF-γ	-	THP-1 macrophages	↓ ICAM-1; ↓ VCAM-1 expression in a dose-dependent manner.	(46)
Lauric acid, Methyl Laurate, Glycerol Monolaurate	NF-κB	RAW 264.7	↔ NF-κB minimal effect; ↑ activation of TLR4 via TLR4-MD2 complex.	(20)
Myristic acid	–	Adipose tissue macrophages	↓ Body weight; ↑ Visceral adipose tissue mass; ↑ Plasma insulin; ↑ AT inflammation; ↑ Resistin.	(47)
Capric acid	TLR4/NF-κB signaling	RAW 264.7 macrophages	↓ Body fat, ↓ lipid profiles, ↓ inflammatory cytokines, ↓ TLR4, ↓ TNF-α.	(51)

Stearic acid influences CD11c expression through nuclear retinoid acid receptors and epidermal fatty acid-binding protein (E-FABP), both of which are critical in driving macrophage differentiation towards a pro-inflammatory CD11+ phenotype (34). Furthermore, stearic acid influences key metabolic pathways by inhibiting the AMP-activated protein kinase (AMPK) phosphorylation and sirtuin 1 (SIRT1) dependent signaling, two central regulators of cellular energy homeostasis (43). The activation of MAPK and SIRT1 has been implicated in metabolic programming, promoting mitochondrial biogenesis, lipid oxidation, and anti-inflammatory responses, thereby playing a protective role against obesity-induced insulin resistance and chronic inflammation (44).

There are instances where SFAs exhibit context-dependent or even contradictory effects, as illustrated by lauric acid. In insulin-resistant THP-1 macrophages, lauric acid enhanced mitochondrial biogenesis and glucose uptake at concentrations of 5-50 μM (24 h), thereby increasing the expression of PGC-1α, TFAM, GLUT1, and GLUT-3, thereby attenuating insulin resistance and promoting metabolic health (45). In line with these findings, lauric acid has been reported to suppress the expression of adhesion molecules ICAM-1 and VCAM-1 in human macrophages, further supporting its anti-inflammatory potential (46). In contrast, lauric acid activated pro-inflammatory signaling in RAW264.7 macrophages by inducing TLR4 dimerization and its recruitment into lipid rafts with MD-2, a proximal step that initiates NF-κB activation in a manner similar to LPS (20). Available studies do not report explicit cell-viability thresholds for lauric acid. However, in insulin-resistant THP-1 macrophages, micromolar exposures (5-50 μM, 24h) were well tolerated and improved mitochondrial function (45), whereas in RAW264.7 cells, higher doses (~150 μM sodium laurate) rapidly triggered TLR4/MD-2-mediated NF-κB activation (20). These findings suggest that the inflammatory outcome depends on the context due to variations in dosage conditions.

By contrast, myristic acid is associated with increased body weight, adipose tissue inflammation, and insulin resistance, contributing to the metabolic disease progression. This indicates that dietary supplementation with myristic acid exacerbates adipose tissue inflammation and systemic insulin resistance in mice subjected to a high-fat diet (47). The study observed increased macrophage infiltration and elevated expression of pro-inflammatory markers in adipose tissue, alongside higher plasma insulin levels and impaired glucose tolerance (48).

On the other hand, capric acid (10:0) has demonstrated potential metabolic benefits, reducing body fat, improving lipid profiles, and suppressing inflammatory responses through TLR4 receptor and NF-κB signaling (49–51).

These findings underscore the complex and nuanced roles of fatty acids in regulating macrophage function and inflammation. While certain SFAs, such as lauric acid and capric acid, may confer metabolic or anti-inflammatory benefits under defined conditions, others like myristic promote inflammation and metabolic dysfunction. Understanding the specific effects of different fatty acids on macrophage function could inform targeted therapeutic strategies for managing metabolic disorders and inflammation-related conditions.

Activation of macrophages by SFAs, particularly palmitic acid, induces a pro-inflammatory phenotype characterized by elevated production of cytokines such as TNF-α, IL-6, and IL-1β, alongside activation of the NF-κB pathway (17). This state is associated with endoplasmic reticulum stress and generation of reactive oxygen species (ROS), contributing to chronic inflammation and insulin resistance in metabolic diseases (28). SFAs activate receptors such as TLR4, TLR2, and the scavenger receptor CD36, which together trigger downstream signaling pathways including NF-κB, JNK, and p38 MAPK, as well as the NLRP3 inflammasome (22). Additionally, SFA disrupt lipid metabolism by promoting lipid accumulation and enhancing fatty acid oxidation, resulting in low-grade, sustained inflammation when exposure is prolonged. In contrast, macrophage activation by lipopolysaccharide (LPS) occurs exclusively through TLR4, which engages the MyD88 and TRIF signaling pathways, leading to the activation of NF-κB and production of cytokines through a typically acute and transient inflammatory response (23, 28). These differences highlight not only the specific signaling pathways involved but also how the persistence and resolution of the stimulus shape the temporal dynamics of the immune response, influencing whether inflammation becomes chronic (17, 53).

## Impact of unsaturated fatty acids on the function of macrophages

UFAs, distinguished by the presence of one or more double bonds in their hydrocarbon chain, exist either in *cis* or *trans* configurations based on spatial arrangements around the double bond. The presence of *cis* double bonds introduces a structural bend, conferring flexibility to the hydrocarbon chain. This property prevents tight packing within membrane phospholipids, thereby increasing the fluidity of the membrane (54). Dietary inclusion of UFAs has been associated with numerous health benefits, including enhanced cardiovascular health, anti-inflammatory effects, and improved cognitive function (2, 55). Among UFAs, polyunsaturated (PUFAs) and monounsaturated (MUFAs) are particularly recognized for their roles in supporting metabolic and immune functions, with each type offering distinct but complementary health benefits; enhancing tissue repair, and contributing to cardiovascular health by reducing triglycerides and improving endothelial function (56). PUFAs, which contain two or more double bonds, are primarily classified into ω-3 and ω-6 families, whereas MUFAs possess a single double bond, conferring them structural and functional properties distinct from PUFAs. Both play a critical role by activating or suppressing distinct signaling pathways that regulate polarization and inflammatory responses function.

Like SFAs, PUFAs and MUFAs exert varying effects on inflammation and metabolism, with some demonstrating anti-inflammatory benefits while others promote inflammatory responses. These effects depend on the type of fatty acid and its dietary context. For example, ω-3 PUFAs such as eicosapentaenoic acid and docosahexaenoic acid are generally anti-inflammatory (57, 58), whereas ω-6 PUFAs, like arachidonic acid (C20:4) (59), can promote inflammation when consumed excessively. Similarly, MUFAs such as oleic acid (C:18:1) (60) have anti-inflammatory properties, but oxidized MUFAs may contribute to inflammatory responses. Overall, while UFAs are broadly associated with beneficial effects on inflammation and metabolic health, certain PUFAs and MUFAs may exert pro-inflammatory effects under specific conditions.

### Polyunsaturated fatty acids

PUFAs play a critical role in regulating metabolic pathways and are integral components of phospholipid bilayers in cell membranes, where their unsaturated hydrocarbon chains contribute to fluidity and flexibility (54). Functionally, PUFAs serve as precursors for bioactive lipid mediators known as oxylipins, which are generated via enzymatic pathways (e.g., cyclooxygenase [COX], lipoxygenase [LOX], and cytochrome P450 systems) as well as non-enzymatic processes involving free radicals (61). Oxylipins regulate key biological processes, including inflammation, tissue homeostasis, and intercellular communication, by influencing intracellular signal pathways. Importantly, their effects are context dependent. Pro-inflammatory oxylipins such as prostaglandins and leukotrienes, derived from arachidonic acid, amplify inflammation, while resolvins, maresins, and protectins form eicosapentaenoic acid and promote resolution and tissue repair (62).

One key pathway in macrophages is that mediated by the peroxisome proliferator-activated receptor gamma (PPAR-γ), through which PUFAs promote an M2 phenotype, reprogramming immune cells into a reparative state and fostering an anti-inflammatory environment (59). Additionally, PUFAs enhance the cholesterol efflux pathways in macrophages, reducing foam cell formation and exerting anti-atherogenic effects (63, 64).

In macrophages, ω-3 PUFAs facilitate the transition from inflammation to resolution, supporting tissue repair by reinforcing the M2 macrophage state. This effect is mediated through the production of specialized pro-resolving mediators (SPMs), which include resolvins, maresins, and protectins (65). D-series resolvins, derived from eicosapentaenoic acid and docosahexaenoic acid, such as resolvin D1 (RvD1), are potent anti-inflammatory mediators, suppressing the NF-κB signaling pathway, enhancing macrophage phagocytosis, and promoting the clearance of inflammatory cells and debris (66). Maresins and protectins, both synthesized from docosahexaenoic acid, further contribute to inflammation resolution by promoting tissue repair and macrophage-mediated efferocytosis (the clearance of apoptotic cells) (67, 68). Mechanistically RvD1 signals through G-protein-coupled receptors (GPCRs) such as ALX/FPR2 and GPR32, both expressed on macrophages, initiating a coordinated anti-inflammatory signaling cascade that reduces pro-inflammatory cytokine secretion and promote efferocytosis (67, 68).

In addition to the D-series, E-series resolvins derived from eicosapentaenoic acid, most notably resolvin E1 (RvE1), exert complementary effects in resolution. RvE1 was originally characterized in murine peritonitis models and human peripheral blood neutrophils and monocytes, where it inhibits leukotriene-driven inflammation by antagonizing BLT1 while simultaneously engaging ChemR23 (ERV1) to promote efferocytosis and limit neutrophil infiltration (69). In addition to resolvins, several families of specialized pro-resolving mediators (SPMs) demonstrate receptor specificity. Maresin-1 was shown in human macrophages and mouse peritonitis models to engage LGR6 and orchestrate regeneration processes, while protectin PD1 has been associated with GPR37, imparting neuroprotective and pro-resolving properties (70, 71). These pathways collectively illustrate the integration of several PUFA-derived mediators and their corresponding receptors to promote homeostasis, tissue repair, and resolution.

In addition, α-linolenic acid (C18:3), an essential ω-3 PUFA, exhibits antioxidative properties that regulate cellular redox balance (72). By upregulating key enzymes such as superoxide dismutase (SOD) and catalase, α-linolenic acid helps mitigate ROS production, thereby limiting oxidative stress and reducing ROS-induced cellular damage in macrophages (61). This protective mechanism may play a crucial role in preventing chronic inflammation. Moreover, α-linolenic acid influences immune responses by inhibiting the production of pro-inflammatory cytokines (48) and enhancing oxylipin-mediated inflammation resolution (61).

On the other hand, ω-6 PUFAs exhibit distinct pro-inflammatory and anti-inflammatory properties, with their effects on macrophages playing a crucial role in inflammatory processes. For instance, linolenic acid, an essential ω-6 PUFA, enhances macrophage activation and remodels lipid raft composition, thereby promoting key signaling pathways (73). In contrast, conjugated linoleic acid, a group of geometric and positional isomers of linoleic acid (C18:2), impacts adipose tissue inflammation by altering adipocyte differentiation. Conjugated linoleic acid supplementation has been shown to reduce inflammation in obese rats by decreasing pro-inflammatory M1 macrophages and increasing anti-inflammatory M2 macrophages (74). Specifically, the 10,12-conjugated linoleic acid isomer promotes weight loss and increases anti-inflammatory macrophages in adipose tissue, underscoring its therapeutic potential in mitigating obesity-induced inflammation (75).

Similarly, γ-linolenic acid (C18:3), another ω-6 PUFA, exerts context-dependent effects on macrophage responses by exhibiting both pro-inflammatory and anti-inflammatory effects. Evidence shows that γ-linolenic acid can suppress NF-κB and AP-1 activation, thereby inhibiting the expression of inflammatory mediators (76). However, other studies indicate that γ-linolenic acid does not consistently affect cytokines such as IL-6, suggesting that its role may vary depending on the experimental context (77). This dual function highlights the complex interplay of linoleic acid, conjugated linoleic acid, and γ-linolenic acid within inflammatory processes, revealing a dynamic regulation of macrophage activity. γ-linolenic acid illustrates the context-dependent effects of PUFA: in RAW264.7 macrophages, it increases IL-6 secretion and activates NF-κB/AP-1 signaling, hence promoting chronic inflammation (76). Conversely, γ-Linoleic acid can stimulate PPAR-γ in human immune cells, increasing IL-10 production and thereby suppressing pro-inflammatory mediators (15). This highlights that the impact of certain fatty acids on macrophage activity is profoundly influenced by cellular environment, dose, and metabolic state.

The interaction between PUFAs and macrophages is important because it influences inflammatory responses, which has implications for the development of therapeutic strategies targeting inflammatory diseases. Understanding the nuanced effects of different PUFAs on macrophage function may offer important lessons for future PUFA-based therapies aimed at mitigating metabolic and immune disorders. **Table 2** provides an overview of the various types of PUFAs, their dietary sources, and their physiological effects.

**Table 2:** Metabolic pathways of PUFAs in macrophages and their effects.

Fatty acid	Metabolic pathways or associated receptors	Type of macrophages	Main results	Reference
Docosahexaenoic acid	Lipolytic and fatty oxidation pathways	scWAT in obese aged female C57BL/6J mice macrophages	↓ M1 marker CD11c; ↑ M2 marker CD206.	(78)
Docosahexaenoic acid	Inflammatory and anti-inflammatory response by ELOVL2 enzyme	Bone marrow-derived M1/M2 macrophages	↑ M1 markers (iNOS, CD86); ↓ M2 markers (CD206).	(79)
Eicosapentaenoic acid	NLRP3 inflammasome	Human THP-1 monocyte-derived macrophages	↓ IL-1β and IL-18; ↓ inflammasome gene expression.	(57)
Docosahexaenoic acid and eicosapentaenoic acid	Inflammation and insulin resistance	Bone-marrow derived M0, M1, and M2 macrophages	↑ TG levels in M1; TG profiles shift to PUFAs.	(33)
Docosahexaenoic acid	Lipokine pathways	Murine 3T3-L1 adipocytes, human PBMCs, RAW 264.7 macrophages	↓ Novel lipokines (13-DHAHLA); ↓ inflammation.	(58)
Eicosapentaenoic acid	GPR120 receptor	3T3-L1 adipocytes, HFHS diet mouse model	↓ TAK1/TAB1, crown-like structures in adipocytes.	(80)
Eicosapentaenoic acid	GPR119 and GPR120 receptors	Adipose tissue macrophages	↑ Tregs induction by Eicosapentaenoic acid; ↑ ATM-mediated Tregs.	(63)
Eicosapentaenoic acid	GPR120	Adipocytes 3T3-L1 and mice adipose tissue macrophages	Supplementation with Eicosapentaenoic acid alters phenotypes of macrophages from M1 to M2 in the vascular stromal fraction.	(80)
α-linolenic acid	Oxylipin pathways	Human THP-1 cell-derived M1-like macrophages	↓ IL-6, IL-1β, TNF-α; ↑ oxylipins.	(61)
Conjugates linoleic acid, α-linolenic acid	Cholesterol transport pathways	ApoE3L.CETP mice on HFD	↓ Systemic cholesterol; ↓ liver cholesterol levels.	(64)
Medium-chain diacylglycerols	Inflammation	RAW264.7 macrophages and 3T-L1 adipocytes	↓ Phagocytosis, ↓ IL-6, ↓ TNF-α, ↓ COX-1; ↓ iNOS, and ↓ CD80 on cell surfaces in LPS-stimulated macrophages.	(50)
Oleanolic acid (SO1989)	Fatty acid oxidation	HDF-induced obese mice macrophages	↑ M2 polarization; ↓ inflammation, reduced adipose inflammation, and improved metabolic functions.	(81)
Arachidonic acid (C20:4)	PPAR-γ and PGE2	Human THP-1-derived and mouse bone marrow-derived	M2 macrophage polarization inhibition; OXPHOS activation.	(59)
Thromboxane A_2_ derived from arachidonic acid	Thromboxane-TP signaling	Obese mice TP knockout mice macrophages	↑ M1 polarization; ↑ Insulin resistance; ↓ Adipocyte hypertrophy.	(82)
Oleic acid	–	Macrophages form Wistar albino rats	↓ Body weight, TG, cholesterol, TNF-α, IL-6 levels; ↑ HDL; ↑ UPC1, CD137, CD206 expression.	(83)
Oleic acid	Inflammatory regulation	M2 macrophages in mesenteric adipose tissue from C57BL/6 male mice	↑ M2 markers CD206, MGL1, and ARG1.	(84)
Oleic acid	Macrophage dysfunction	Murine J774.2 macrophages	↓ NO secretion; ↓ TNF-α, a potential link to higher cancer and allergy risks in obesity.	(60)
Oleic acid and palmitic acid	TLR2-mediated inflammatory responses	Bone marrow-derived macrophages (BMDM)	↑ Pro-inflammatory gene expression (CXCL1, IL-6, TNF-α); ↓ Cytokine secretion upon LTA stimulation.	(42)
SO1989, a derivative of oleic acid	Macrophage polarization	Macrophages from high-fat-diet-induced obese mice	M1 to M2 balance restoration; ↑ fatty acid oxidation.	(81)
Conjugated linoleic acid	–	Ldrl-/- mice	↑ M2 macrophages; ↓ CD68, improved cholesterol and triglyceride levels; ↓ Atherosclerosis.	(74)
10,12 – Conjugated linoleic acid	–	12-week mice macrophages	↑ Fat oxidation; ↑ M2 macrophages; ↑ Anti-atherosclerotic effects; ↑ Lesion macrophage content.	(75)
Palmitoleate	Inflammation	Bone marrow-derived macrophages (BMDM) from high-fat-fed and low-fat-fed mice	↑ M2 markers; ↑ oxidative metabolism; ↓ pro-inflammatory gene expression (Cxcl1, IL-6, IL12b).	(40)
Palmitoleic acid	Insulin resistance dependent on diacylglycerols	Macrophages of 12-week mice	↓ Macrophage infiltration; ↑ Glucose uptake.	(85)
γ-Linoleic acid	NF-κB, AP-1, PPAR-γ	RAW264.7 macrophages	↑ IL-6 secretion; ↑ IL-10 via PPAR-γ activation (context dependent).	(76)

## Impact of monounsaturated fatty acids on macrophages’ function

MUFAs, characterized by a single double bond, typically in the *cis* configuration, are abundant in plant-based oils such as olive, canola, and avocado oil. The Mediterranean diet, rich in MUFAs, has been associated with health benefits, including cardiovascular protection, anti-inflammatory effects, and improved lipid profiles. In this diet pattern, approximately 60% of total fat intake is derived from MUFAs, which have been shown to lower blood pressure, enhance glucose metabolism, and reshape gut microbiome, thereby reducing the risk of coronary artery disease (56).

Among MUFAs, oleic acid —a major component of olive oil—exhibits notable anti-inflammatory effects and improves insulin sensitivity by suppressing TLR4 signaling in macrophages (60, 86). This suppression downregulates downstream inflammatory pathways, particularly those mediated by NF-κB and MAPK kinases, leading to a reduction in pro-inflammatory cytokines such as IL-6 (42). Consequently, this suppression contributes to an overall anti-inflammatory response. Furthermore, oleic acid promotes macrophage polarization toward the M2 phenotype by activating PPAR-γ, favoring a reparative and anti-inflammatory state (83, 84). This activation not only enhances the expression of genes involved in the differentiation of insulin-sensitive adipocytes but also supports inflammation resolution by reducing macrophage infiltration into adipose tissue, a key driver of obesity-associated inflammation.

In addition to its effects on macrophage polarization, oleic acid (C18:1 cis-9) and other MUFAs also mitigate oxidative stress by enhancing the activity of antioxidant enzymes such as superoxide dismutase (SOD), glutathione peroxidase (GPx), and catalase (72). This mechanism protects cells from oxidative damage, further facilitating inflammation resolution. Importantly, PPAR-γ activation by MUFAs not only reduced NF-κB signaling but also synergistically enhanced antioxidant enzyme activity, creating a dual mechanism that simultaneously combats oxidative stress while promoting anti-inflammatory macrophage function (36). This combined action helps reduce macrophage accumulation in adipose tissue, thus alleviating inflammation associated with obesity and metabolic disorders (87).

However, not all MUFAs exhibit the same effects. Erucic acid (C22:1), found in yellow mustard, has been reported to act as a natural inhibitor of PPAR-γ transcriptional activity, improving insulin sensitivity and reducing macrophage infiltration into adipose tissue (88). This effect contrasts with the typical benefits of PPAR-γ activation, seen with oleic acid and ω-3 PUFAs, where PPAR-γ activation promotes an anti-inflammatory M2 macrophage phenotype and contributes to inflammation resolution (89). These findings underscore that MUFAs are a heterogeneous group of fatty acids, with effects that depend on the specific fatty acid, its interaction with PPAR-γ, and its metabolic context. The differing impact of MUFAs on macrophage function illustrates the importance of a more comprehensive understanding of how various MUFAs may either upregulate or downregulate specific inflammation pathways.

A key consideration in the dual role of PPAR-γ in macrophage function is that while PPAR-γ activation reduces inflammatory cytokine production in M1 macrophages and potentiates M2 activation, it may also impair full M2c polarization, a macrophage subtype critical for achieving an inflammatory-resolution state (90). This suggests that chronic PPAR-γ activation, as observed in obesity or metabolic syndrome, may hinder complete inflammation resolution. In this context, PPAR-γ antagonism during monocyte-to-macrophage differentiation has been proposed as a strategy to promote M2c-like polarization, helping restore anti-inflammatory conditions in chronic inflammatory settings (89, 90). These findings emphasize the complex-dependent effects of PPAR-γ on macrophage function, which points to the therapeutic benefits of selectively targeting specific PPAR-γ isoforms to either enhance or inhibit macrophage polarization and metabolic pathways depending on context.

While many MUFAs (such as oleic acid) are generally anti-inflammatory and promote M2 polarization, nervonic acid (C24:1) exhibits a more complex role in inflammation. Nervonic acid has been studied for its effects on immune function in RAW 264.7 macrophages, where it has been found to activate NF-κB signaling, leading to the increased production of pro-inflammatory cytokines (e.g., TNF-α and IL-6) as well as enhanced nitric oxide (NO) and ROS generation in concentrations of 12.5 μM or higher (91). These findings suggest that nervonic acid exerts dose-dependent effects on macrophage function, reinforcing that not all MUFAs follow the same pattern regarding macrophage polarization and inflammation. Further research is needed to fully elucidate the mechanisms of nervonic acid in different physiological contexts and explore its potential role in macrophage activity (72).

In animal models, such as in JCR rats (a model of obesity), vaccenic acid (C18:1 trans-11), a precursor to trans fats derived from ruminants, has been shown to suppress pro-inflammatory lymphocyte activity and improve the inflammatory profile of mesenteric lymphocytes (92). These findings point out the importance of MUFAs in regulating macrophage activity and inflammation, particularly in obesity-related metabolic disorders (see **Table 3**). Additionally, these results highlight the complexity and diversity of MUFA effects on immune function, demonstrating that MUFAs cannot be generalized into one anti-inflammatory category.

**Table 3:** Summary of MUFAs effects on metabolic and immunological pathways.

Fatty acid	Metabolic pathways or associated receptors	Type of macrophages	Main results	Reference
Eicosenoic acid	PPAR-γ	Macrophages from KK-Ay obese/diabetic mice	↓ PPAR-γ transcriptional activity and adipocyte differentiation; ↓ insulin resistance; ↓ macrophage infiltration.	(88)
Nervonic acid	NF-κB signaling pathway via TLR4 pathway	RAW264.7 macrophages	↑NO, TNF-α, IL-1β, IL-6, ROS production; ↑ iNOS, TNF-α, IL-1β, IL-6 mRNA expression; ↑ TLR4 protein expression; ↑ NF-κB phosphorylation.	(91)
Vaccenic acid	–	Macrophages from JCR: LA -cp rats	Lean rats: ↓ CD45RC+ helper cells, ↓ IL-2, IL-10, and TNF-α production. Obese rats: Normalized MLN IL-2, TNF-α production, and ↓CD45RC+ helper cells.	(92)

## Concluding remarks

**Figure 2:**
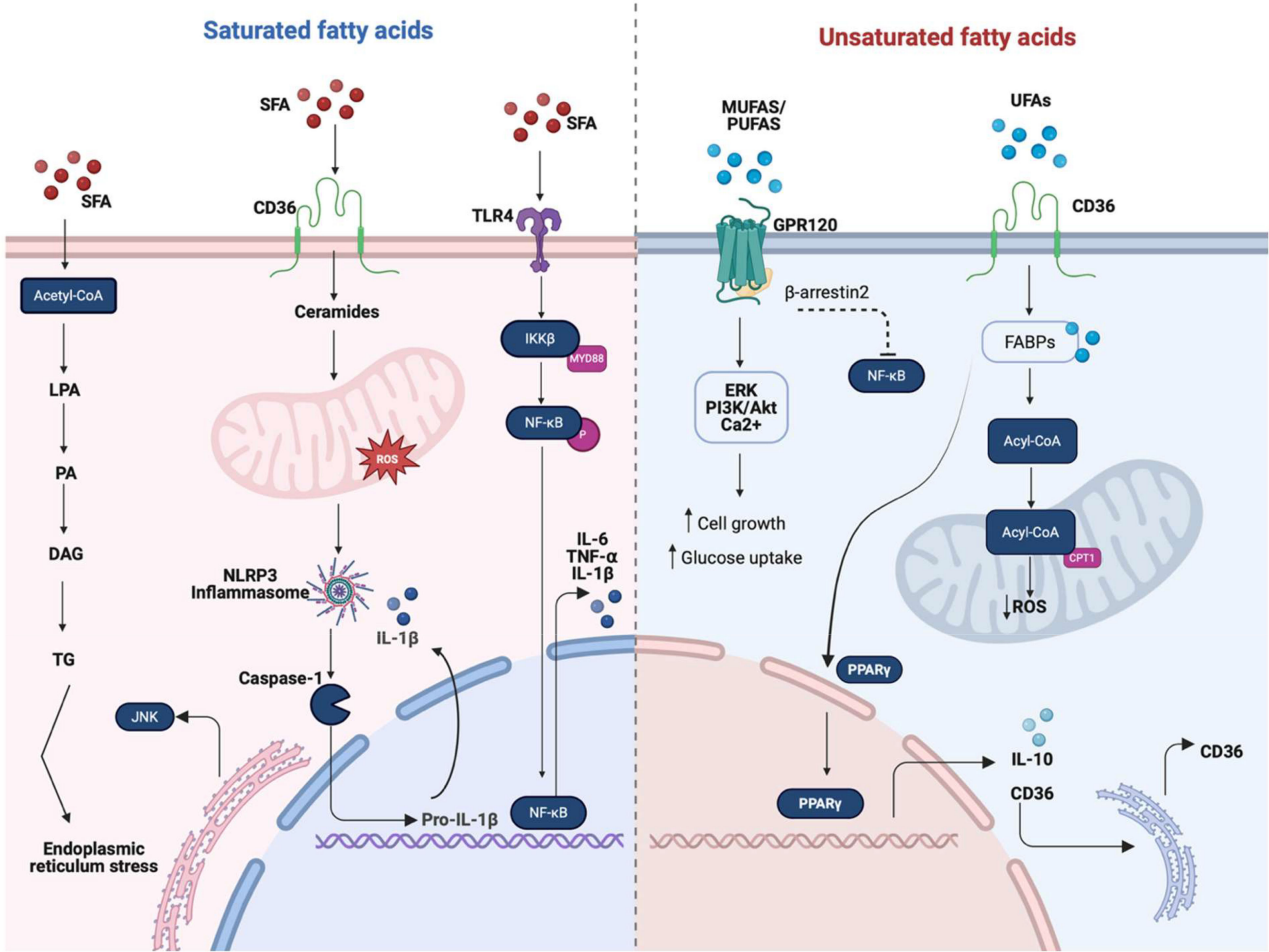
Overview of the main macrophage activation pathways induced by saturated (SFAs) and unsaturated fatty acids (UFAs, including MUFAs and PUFAs). Fatty acids regulate macrophage function by acting as both metabolic substrates and signaling molecules that shape inflammatory responses. SFAs are metabolized into triglycerides (TGs), contributing to endoplasmic reticulum (ER) stress and the activation of pro-inflammatory pathways involving CD36, ceramides, and reactive oxygen species (ROS), which trigger the NLRP3 inflammasome. This cascade promotes inflammation by disrupting membrane fluidity and enhancing TLR4-mediated NF-κB signaling, leading to the production of proinflammatory cytokines such as IL-6, TNF-α, and IL-1β. In contrast, UFAs exert anti-inflammatory and pro-resolving effects. They activate GPR120, which engages multiple downstream pathways, including ERK, PI3K/AKT, and Ca^2+^ signaling, that collectively enhance glucose uptake and cell growth. UFAs also signal through CD36 and fatty acid-binding proteins (FABPs), which transport fatty acids to mitochondria for conversion to acetyl-CoA. Acetyl-CoA enters β-oxidation, facilitated by carnitine palmitoyl transferase 1 (CPT1), thereby enhancing mitochondrial respiration and reducing ROS levels. This metabolic shift supports the activation of PPAR-γ and the production of IL-10, reinforcing an M2 phenotype through feedback to CD36. Together, these pathways highlight the dual immunometabolic roles of fatty acids in macrophage polarization and suggest potential targets for therapeutic intervention in metabolic inflammatory diseases. Created in https://BioRender.com.

The intricate interplay between fatty acid metabolism and immune cell function unveils a complex regulatory network that impacts both physiological and pathological processes. As illustrated in **Figure 2**, the metabolic activation of macrophages in response to different types of fatty acids reveals divergent functional outcomes. Fatty acid oxidation plays a critical role in promoting alternative and regulatory macrophage phenotypes, particularly the M2 phenotype, which is involved in tissue remodeling, wound healing, and anti-inflammatory cytokine production. Key metabolic regulators, such as PPAR-γ and AMPK, enhance fatty acid uptake and oxidation in M2 macrophages, supporting their anti-inflammatory and reparative functions (43, 59).

In contrast, *de novo* lipogenesis and fatty acid synthesis are closely associated with the pro-inflammatory M1 macrophage phenotype, which is related to rapid clonal expansion and cytokine production. This metabolic pathway supplies essential lipids for membrane biogenesis and biosynthesis of signaling molecules, such as inflammatory eicosanoids derived from arachidonic acid. The metabolic shift toward glycolysis in M1 macrophages is further regulated by sterol regulatory element-binding protein 1 (SREBP1), which ensures a continuous supply of substrates for fatty acid synthesis to sustain the heightened metabolic demands of inflammation (17).

Our revision highlights the distinct roles of SFAs, MUFAs, and PUFAs in shaping macrophage behavior and function. SFAs activate pattern recognition receptors, particularly TLR4, leading to NF-κB activation and subsequent production of inflammatory cytokines like TNF-α and IL-6. Chronic exposure to SFAs has been implicated in the development of metabolic inflammation and insulin resistance (19). In contrast, PUFAs serve as precursors for SPMs and inhibit NF-κB, thereby reducing pro-inflammatory cytokines production (58). It is important to note that exceptions within each fatty acid class have been reported in the literature, and readers are referred to the previous sections for further details. MUFAs, such as oleic acid, generally exert anti-inflammatory effects by increasing membrane fluidity, attenuating TLR signaling, and promoting oxidative metabolism (84). Moreover, MUFAs have been shown to counteract some of the proinflammatory actions of SFAs, thereby improving insulin sensitivity and reducing macrophage activation.

It is critical to recognize that a significant portion of the current evidence establishing a connection between macrophage polarization and fatty acids derived from *in vitro* studies that employ primary or immortalized cell culture systems. Despite the fact that these models offer mechanistic insights into lipid-immune interactions, they are unable to completely approximate the *in vivo* environment, which is characterized by the critical roles of tissue microenvironment, systemic metabolism, and immune crosstalk. Consequently, it is critical to ascertain the translational relevance of these findings by conducting studies in animal models. Ultimately, these findings need to be validated in human models. For instance, research conducted in mouse models with high-fat diets have shown that macrophage phenotypes in adipose tissue and liver are adjusted by dietary fatty acids (6, 85). Likewise, the *in vivo* validation of fatty acid-immune interactions was underscored by the alteration of mesenteric lymphocyte function in obese JCR rats by vaccenic acid supplementation (92). The integration of these models will be essential for bridging the knowledge gap between cellular discoveries and clinical applications.

In conclusion, the multifaceted interplay between fatty acids and macrophage function represents a compelling nexus with significant implications for human health. As research continues to unravel the complexities of these interactions, the potential to harness fatty acids as therapeutic agents for managing inflammatory and metabolic diseases emerges as a promising avenue for future research and clinical interventions. This review offers a comprehensive synthesis of current knowledge and underscores the need for further investigation into the intersection of fatty acid metabolism and macrophage biology, paving the way for innovative therapeutic strategies and translational applications.
